# A Concise and Comprehensive Description of Shoulder Pathology and Procedures: The 4D Code System

**DOI:** 10.1155/2012/930543

**Published:** 2012-12-04

**Authors:** Laurent Lafosse, Tom Van Isacker, Joseph B. Wilson, Lewis L. Shi

**Affiliations:** ^1^Alps Surgery Institute, Clinique Generale, 4 Chemin Tour la Reine, 74000 Annecy, France; ^2^Department of Orthopaedics, AZ Sint-Lucas, Sint-Lucaslaan 29, 8310 Brugge, Belgium; ^3^Triangle Orthopaedics Associates, 120 William Penn Plaza, Durham, NC 27704, USA; ^4^Department of Orthopaedics, University of Chicago, 5841 S. Maryland Avenue, Chicago, IL 60637, USA

## Abstract

*Background*. We introduce a novel description system of shoulder pathoanatomy. Its goal is to provide a comprehensive three-dimensional picture, with an additional component of time; thus, we call it the 4D code. *Methods*. Each line of the code starts with right versus left and a time designation. The pillar components are recorded regardless of pathology; they include subscapularis, long head of biceps tendon, supraspinatus, infraspinatus, and teres minor. Secondary elements can be added if there is observed pathology, including acromioclavicular joint, glenohumeral joint, labrum, tear configuration, location and extent of partial cuff tear, calcific tendonitis, fatty infiltration, and neuropathy. *Results*. We provide two illustrative examples of patients which show the ease and effectiveness of the 4D code. With a few simple lines, significant amount of information about patients' pathology, surgery, and recovery can be easily conveyed. *Discussion*. We utilize existing validated classification systems for parts of the shoulder and provide a frame work to build a comprehensive picture. The alphanumeric code provides a simple language that is universally understood. The 4D code is concise yet complete. It seeks to improve efficiency and accuracy of the communication, documentation, and visualization of shoulder pathology within individual practices and between providers.

## 1. Introduction

 There are numerous classification systems in the literature that define various pathologic findings associated with the shoulder [1–8]. Individually each of these classifications is accurate and useful in describing a specific portion of the shoulder anatomy or pathoanatomy. Currently, no description system exists to convey a comprehensive three-dimensional image of the shoulder in a concise and reproducible format. In this paper, we introduce a description system to fill this void. The 4D code, as we call it, incorporates a complete three-dimensional picture of the shoulder with the additional component of time. It seeks to improve efficiency and accuracy of the communication, documentation, and visualization of shoulder pathology within individual practices and between treating surgeons.

 As the knowledge and ability of a specialty expand and improves, it is a natural progression that the classification systems utilized by the profession must evolve. Historically, the rotator cuff was visualized as a single pathologic entity, and classification systems described them as such [9]. These prior systems were sufficient for the knowledge of that era. With time, our understanding of shoulder anatomy and physiology has evolved. The rotator cuff is comprised of structurally and functionally distinct components which work synergistically to achieve full function of the shoulder. Goutallier exposed the importance of the status of the muscle, and implications of fatty infiltration when evaluating and treating rotator cuff disorders [10, 11]. Warner has made us aware of the role the suprascapular nerve in the function of the rotator cuff and its causal relationship to various shoulder problems [12, 13]. This expansion of knowledge requires a parallel improvement and update in the method of classification utilized. In addition to the rotator cuff, other structures need to be considered simultaneously in order to fully achieve a global view of the shoulder, including the acromioclavicular (AC) joint, the glenohumeral joint, the long head of biceps tendon, the labrum, and associated nerves. Each of these structures and their interactions directly affect the ultimate integrity and performance of the shoulder complex as a whole.

 Classification systems for tears of the rotator cuff tendon have been proposed based upon specific characteristics such as size of the lesion, number of tendons involved, and the reparability of the tear [1–8, 14, 15]. In addition to these broader-based classifications, specific classification systems focusing on individual tendons have also been proposed. Patte developed a classification for lesions of the supraspinatus, infraspinatus and teres minor, Lafosse for tears of the subscapularis, and Curtis and Ellman for partial rotator cuff tears [2, 4, 7].

 Individually, these rotator cuff tear classification systems do provide information about the condition of the patient's shoulder, but are limited in their description of the shoulder as a whole. In addition, none of these systems provides any information about the status of the associated joints, ligaments, tendons, and nerves; their concomitant assessment and treatment are important in optimizing patients' outcome. The limitations of these current classification systems mandate the development of a new, concise, and expandable classification system that covers all rotator cuff tendon pathology, and is inclusive of the surrounding structures of the shoulder. The 4D code was developed with these explicit goals. 

## 2. Methods

 The aim of the 4D code is to provide a logical method of describing shoulder pathology using universal numbers and letters. The 4D code seeks to provide a complete description of the shoulder in a concise format, without sacrificing detail, and allows a practitioner to glean the pertinent information in regards to preoperative, operative, and postoperative condition of the shoulder. This allows the appropriate treatment protocols to be adjusted and optimized for each individual patient in an efficient and comprehensive manner.

 The 4D code is one or multiple lines of alpha-numeric text, each line denoting the condition of the shoulder at one point in time. We introduce our methodology by dividing the 4D code into its fundamental elements and secondary elements.

### 2.1. The Fundamental Elements

**Figure 1:**
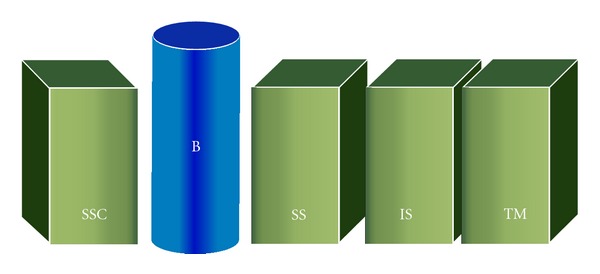
The pillar components of the 4D code. Starting from anterior aspect of the shoulder, the pillar components are subscapularis (SSC), long head of biceps tendon (B), supraspinatus (SS), infraspinatus (IS), and teres minor (TM).

The fundamental elements of the 4D code start with the shoulder sidedness and the time code. Next are the pillar components (Figure 1). These represent anatomical structures of the shoulder which serve as important scaffolds for the 4D code. Starting from the anterior aspect of the shoulder and moving posteriorly, the pillar components include: subscapularis, long head of biceps tendon, supraspinatus, infraspinatus, and teres minor. These structures form the foundation of the shoulder and are always described, regardless whether they have pathology or not. The pillar components have the greatest influence on symptoms, surgical decisions, and patient's function. 

**Figure 2:**
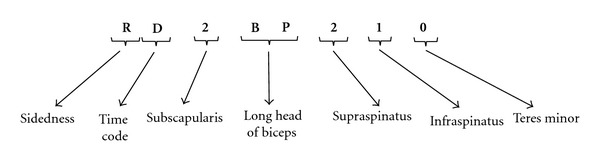
An example of a 4D code with its fundamental elements.

An example of the 4D code which includes the fundamental elements is shown in Figure 2. We will discuss components of this, including sidedness, time code, and all of the pillar components.

#### 2.1.1. Sidedness

 The first letter is the sidedness of the shoulder. An “R” or “L” will start all lines of 4D code, so there is consistency in the reporting of the shoulder of interest of a given patient.

#### 2.1.2. Time Code

**Figure 3:**
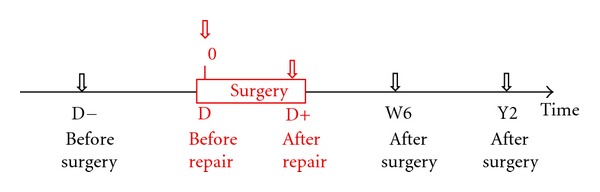
Time code: Time as the fourth dimension in the 4D code system. D− denotes before surgery; D denotes day of surgery prior to repair; D+ denotes immediately after repair. Additional time points can be described, such as D+6W denotes the 6 weeks postoperative visit.

 The second fundamental element is the time code (Figure 3). This allows the pathology of the shoulder to be correlated to a specific time in the treatment time-line.

- D− —before surgery: the code that follows is the condition of the shoulder based on history, examination, radiological, and other studies.
- D—day of surgery, prior to any repair: this includes the important information gathered from diagnostic arthroscopy. The code may be different from before surgery (time D−) due to evolution or clarification of pathology.
- D+ —end of surgery: this presents the condition of the shoulder once repair is complete.
- D+ time—a certain time after surgery: D+6W would represent the time point of 6 weeks postoperatively. M (months) and Y (years) can also be used.

#### 2.1.3. Subscapularis

 The first of the pillar components describes the condition of the subscapularis tendon. We use the Lafosse classification system, which is based on tear size and location, humeral head concentricity, and degree of fatty infiltration (Table 1) [4].

#### 2.1.4. Long Head of Biceps Tendon

 The 4D code documents the condition of the long head of biceps tendon into the following categories:

- BN—normal;
- BP—pathological;
- BA—absent or tenotomized;
- BT—tenodesed.

#### 2.1.5. Supraspinatus, Infraspinatus, and Teres Minor

**Figure 4:**
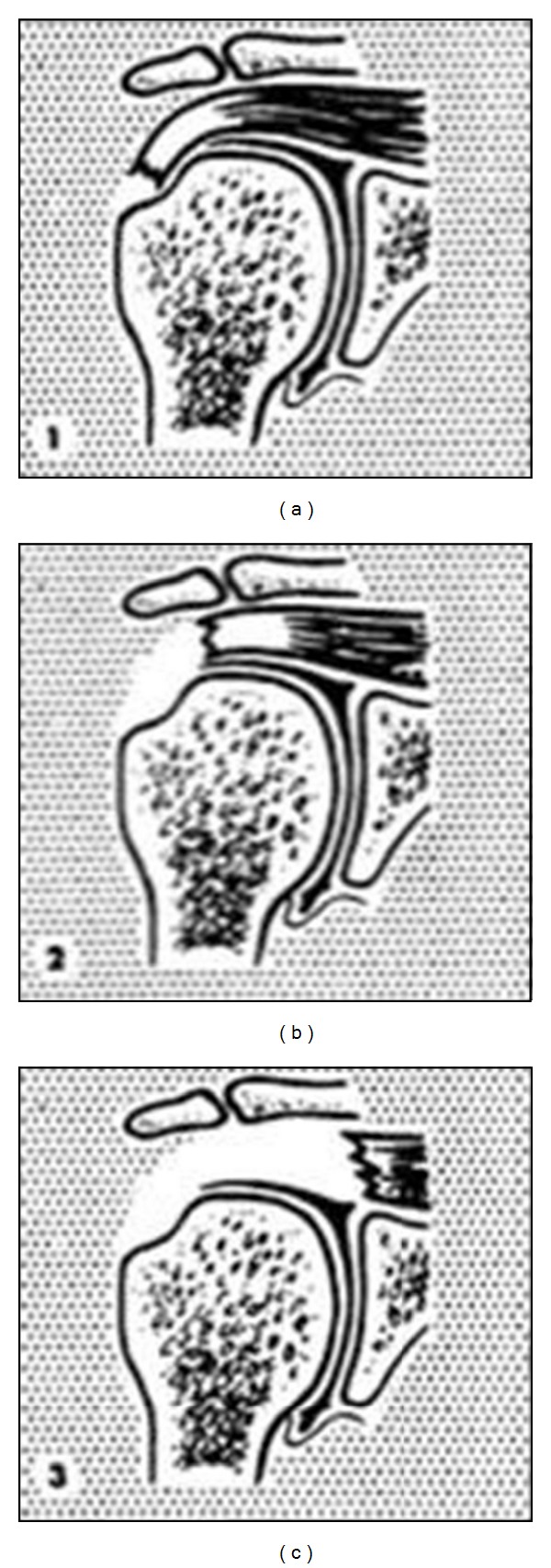
Patte classification system is used to describe tears of the supraspinatus, infraspinatus, and teres minor tendons in the coronal plane [7]. Stage 1: proximal stump is close to its bony insertion. Stage 2: proximal stump at center of humeral head. Stage 3: proximal stump at level of the glenoid.

 To assess the tear in the coronal plane, the Patte classification system is used to describe the supraspinatus, infraspinatus, and teres minor tendons (Figure 4) [7]. Stage 1 describes a tear with minimal retraction; stage 2 describes retraction to center of humeral head; stage 3 describes retraction to glenoid. A “0” is assigned for normal tendons.

 Putting the fundamental elements of the 4D code together, the example in Figure 2 (RD 2 BP 2 1 0) describes the condition of a right shoulder after surgical examination but prior to any intervention. The shoulder has a complete lesion of the superior third of subscapularis (Type 2). The long head of biceps tendon is pathological (BP). Supraspinatus tendon is torn and retracted to top of humeral head (stage 2). Infraspinatus tendon is torn with minimal retraction (stage 1). Teres minor tendon is normal.

### 2.2. The Secondary Elements

**Figure 5:**
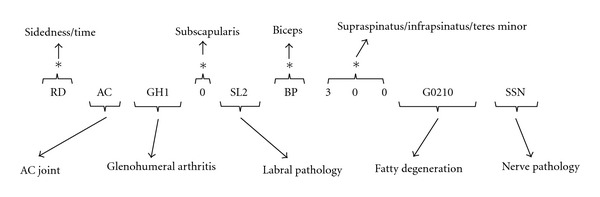
An example of a 4D code with both fundamental elements and secondary elements. Fundamental elements are denoted by (*). The secondary elements include descriptions of AC joint pathology, glenohumeral arthritis, labral pathology, fatty infiltration, and neuropathy.

The secondary elements expand the descriptive capability of the 4D code system. They add to the shoulder sidedness, time code, and the pillar components. Unlike the fundamental elements which appear with every line of 4D code, the secondary elements are documented only if there is pathology. These structures and conditions include: the acromioclavicular (AC) joint arthritis, glenohumeral joint arthritis, labral pathology, fatty infiltration, and any associated nerve pathology. Additional description of a rotator cuff tear may be added, including shape of a tear, bursal- versus articular-sided, and presence of calcific tendonitis.  Figure 5 shows an illustrative line of 4D code which includes all of the secondary elements.

#### 2.2.1. Acromioclavicular Joint

 If there is presence of AC joint disease, this can be added to the 4D code as a secondary element. We choose to place the following code immediately after the time designation:

- AC—presence of AC joint arthritis;
- ACR—status post AC joint resection.

#### 2.2.2. Glenohumeral Arthritis

 The presence of glenohumeral osteoarthritis is documented using the Samilson classification [8]; it is based on the size of the inferior humeral head osteophyte (Table 2). In the 4D code, we use “GH” followed by the Samilson stage to denote glenohumeral arthritis; this is placed before the subscapularis code. 

#### 2.2.3. Labrum

 With superior labral pathology, we use Snyder's classification [16] of the superior labrum anterior posterior (SLAP) tears, later expanded by Maffet et al. [17] (Table 3). The remainder of the labrum can be document using the nomenclature of a clock face. In the 4D code, the following designations are used for labral pathology, placed in the code between subscapularis and biceps tendon:

- SL*x*: type *x* SLAP lesion;
- SLD: debridement of SLAP lesion;
- SLR: SLAP repair;
- L*x*-*y*: labral detachment from *x* to *y* o'clock, for example, L2–6 is a Bankart lesion from 2 to 6 o'clock;
- LD: labral debridement;
- LR: labral repair.

#### 2.2.4. Tear Configuration

 The shape of the rotator cuff tear can be incorporated into the 4D code. Full thickness tears have been described as U-shaped, V-shaped, or L-shaped in the literature. We use the following designation immediately before the tendon it is associated with:

- U: U-shaped tear;
- V: V-shaped tear;
- AL: anterior L-shaped tear; anterior corner detachment;
- PL: posterior L-shaped tear; posterior corner detachment.

#### 2.2.5. Partial Thickness Cuff Tear

 Partial thickness tears of the supraspinatus, infraspinatus, and teres minor are documented in the 4D code according to the Ellman classification (Table 4) [2]. This would take place of the Patte classification if the cuff tear is partial-thickness. It would be a letter designation for location of the tear followed by a number indicating the Ellman grade. Partial tears of the subscapularis tendon are included in the aforementioned Lafosse classification and do not require additional designation:

- A*x*: articular-sided tears with Ellman grade *x*
- B*x*: bursal-sided tears with Ellman grade *x*
- I: interstitial tears

#### 2.2.6. Calcific Tendonitis

 Calcium deposit in the rotator cuff tendon can cause exquisite pain and is an important pathology in the shoulder. If calcific tendonitis is present, the letter C can be placed in the 4D code for the particular tendon involved. 

#### 2.2.7. Fatty Infiltration of Rotator Cuff

The presence of fatty infiltration in the rotator cuff muscle provides additional information regarding chronicity of the tears and has significant impact on surgical decision making and the potential for return of function [11]. Fatty infiltration is documented in the 4D code according to the Goutallier classification (Table 5) [10]. This component being a secondary element, it is documented only if there is presence of fatty infiltration. A letter “G” is placed after the pillar components and is followed by four numbers, corresponding to fatty infiltration of subscapularis, supraspinatus, infraspinatus, and teres minor, respectively.

#### 2.2.8. Nerve Pathology

 If any neuropathy is present around the shoulder based on history, examination, imaging, and neurodiagnostic studies, this can be designated in the 4D code:

- SSN—suprascapular nerve injury;
- SSNR—status post suprascapular nerve release;
- AXN—axillary nerve injury;
- LTN—long thoracic nerve injury.

Table 6 summarizes the fundamental and secondary elements of the 4D code, including its possible codes and corresponding descriptions.

## 3. Results

We present two patients from our practice as illustrative examples of the utility of the 4D code.

### 3.1. Patient 1

 A 64-year-old lady initially presented in consultation with right shoulder pain. Her symptoms had started insidiously one year earlier. She did not improve with nonoperative treatments and elected to undergo rotator cuff repair. She did well initially; however, she sustained a fall 2 years after surgery and has returned to the office. Using the 4D code, her clinical picture can be summarized as follows.

 Line 1: RD− 1 BP 2 0 0.
 Line 2: RD 2 BP 2 1 0.
 Line 3: RD+ 0 BT 0 0 0 SSNR.
 Line 4: RD+6W 0 BT 0 0 0 SSNR.
 Line 5: RD+2Y 0 BT B1 0 0 SSNR.

From these five lines of code, a significant amount of information is conveyed. Line 1 portrays the condition of the right shoulder at time of consultation (D−). Based on examination and three-dimensional imaging, there is partial tear of the upper subscapularis tendon, biceps tendon is diseased, and supraspinatus tendon is torn and retracted to the level of humeral head. Line 2 shows the condition of the shoulder on the day of surgery after diagnostic arthroscopy (time D); superior subscapularis tendon is noted to be completely torn, and in addition to supraspinatus tear, infraspinatus is torn as well without retraction. In line 3, we see the extent of the surgical interventions (time D+), which include tenodesis of the long head of biceps, successful anatomical repair of all rotator cuff tendons, and release of the suprascapular nerve. At the six-week-followup (time D+6W), line 4 shows that the repair is intact. After patient sustained a fall two years later (time D+2Y), examination and additional imaging shows (line 5) that the cuff repair remains intact, but there is a bursal-sided partial tear of the supraspinatus tendon.

 No additional secondary elements are listed because this patient had no AC joint or glenohumeral joint arthritis, labrum was normal, and there is no fatty infiltration of the rotator cuff muscles. 

### 3.2. Patient 2

**Figure 6:**
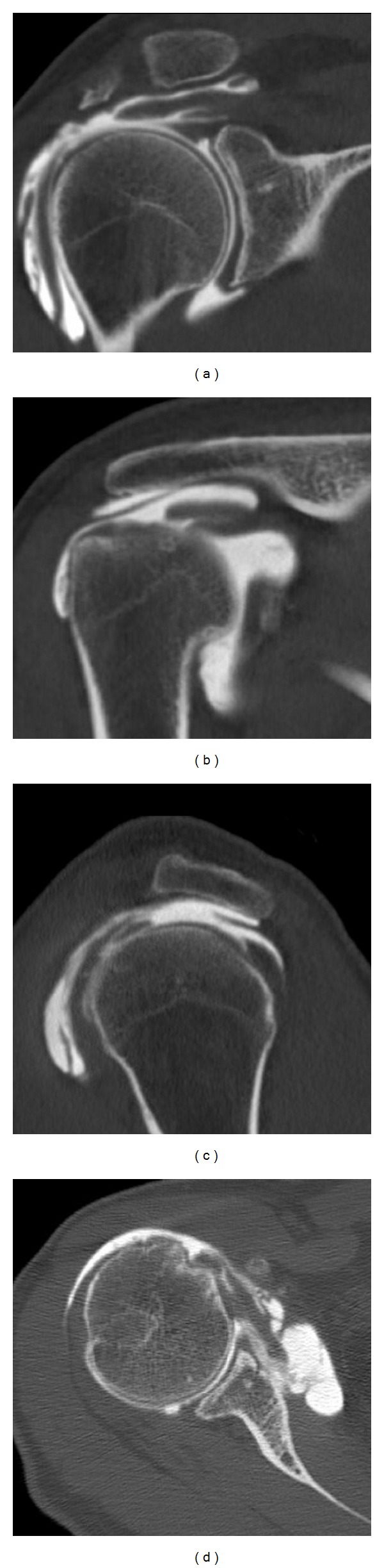
Patient 2 had the following CT arthrogram at time of presentation. It confirmed the exam findings of torn subscapularis, supraspinatus, and infraspinatus. Biceps tendon was diseased.

This patient is a 58-year-old male with a long history of dominant right shoulder pain and functional limitation. At the patient's initial visit, he complained one year of worsening right shoulder pain, atraumatic in onset. He had failed to improve despite physical therapy and one subacromial corticosteroid injection. His examination showed a torn subscapularis tendon from positive Lift-off and Bear-hug tests. He also had weakness of the supraspinatus and infraspinatus muscles. He had bicipital groove pain but no AC joint tenderness. His CT arthrogram confirmed and added to the exam findings (Figure 6). There was complete tear of the upper portion of subscapularis, diseased/subluxed biceps tendon, and moderately retracted supraspinatus and infraspinatus tendons. No obvious fatty infiltration was visualized. Based on these information, we recorded the following 4D code.

**Line 1: RD− 2 BP 2 2 0**

**Figure 7:**
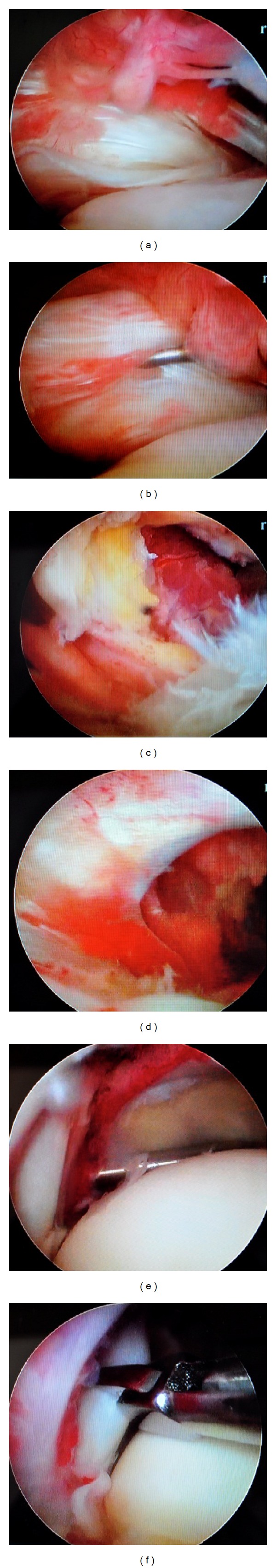
Patient 2's diagnostic arthroscopy documentation. (a) Initial inspection shows an absent medial biceps pulley and a tear of at least the upper one-third of the subscapularis tendon. (b) There is intratendinous extension of the tear as demonstrated by the arthroscopic probe. (c) Inferiorly, the upper two-thirds of the lesser tuberosity is exposed after debridement. There is significant fraying of the dislocated biceps tendon on the right-hand side. There is also the “comma sign” from the medially retracted anterior-superior rotator cuff tear. (d) This is the articular view of the grade 2 supraspinatus tear retracted to level of the humeral head. (e) This is a lateral view of the exposed humeral head with the grade 2 infraspinatus tear. (f) Infraspinatus tendon has delaminated into two layers.

The patient was indicated for surgery, and photographic documentation was done during diagnostic portion of the arthroscopy (Figure 7). It confirmed initial findings with the exception that the subscapularis tear was more significant, affecting the upper two-thirds of the tendon attachment, making it a Lafosse type 3 tear. The second line of this patient's 4D code reflected the changes.

**Line 2: RD 3 BP 2 2 0**

**Figure 8:**
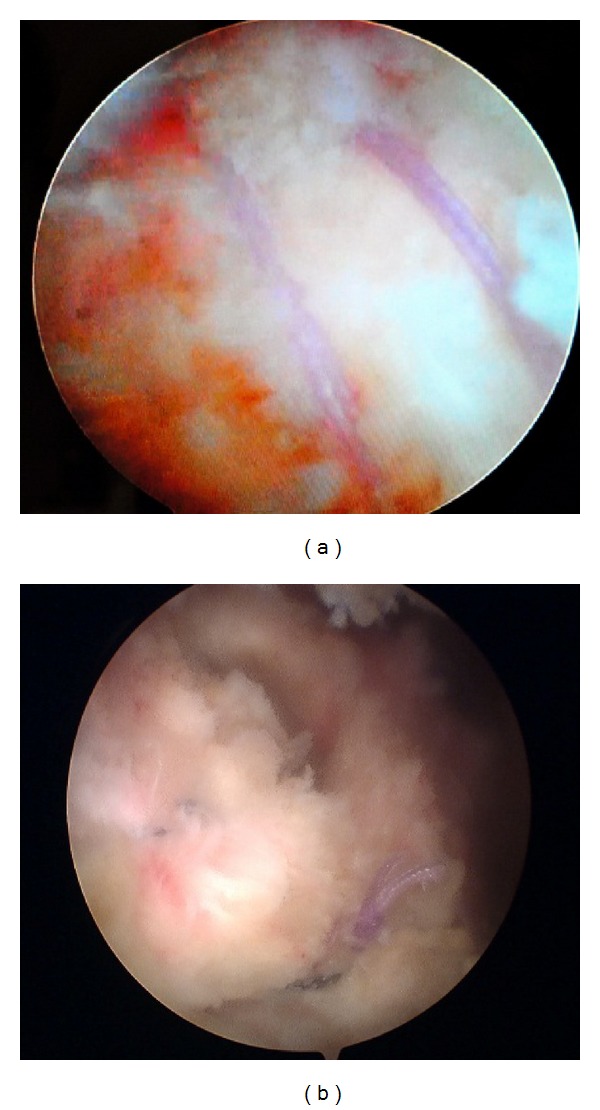
Patient 2's final arthroscopy pictures documenting rotator cuff repair. (a) The posterior cuff is restored anatomically to the footprint of the greater tuberosity. (b) There is an anatomic repair of the supraspinatus and subscapularis tendons with an open rotator interval.

The pathology was addressed with an anatomic, arthroscopic repair of the rotator cuff, and arthroscopic proximal biceps tenodesis. Final pictures are shown in Figure 8. Line 3 documented the status of the shoulder at the conclusion of the surgical intervention.

**Line 3: RD+ 0 BT 0 0 0**

**Figure 9:**
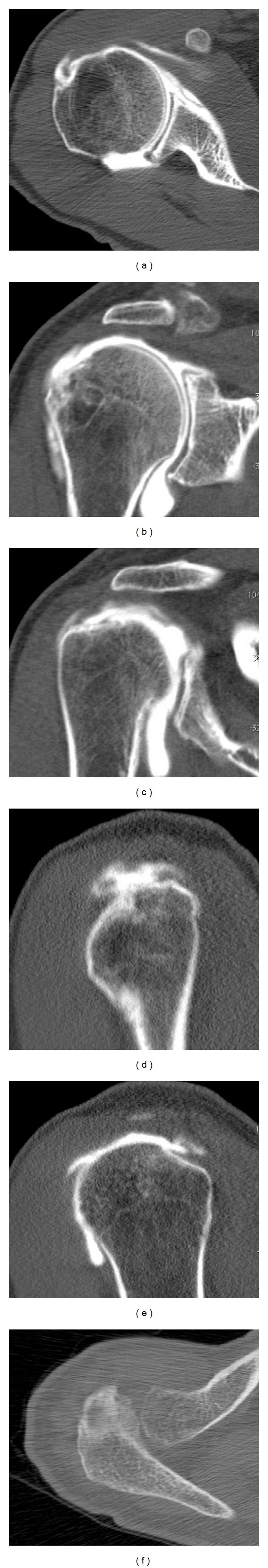
Patient 2's 3-year-followup CT arthrogram. (a) The subscapularis tendon repair and the tenodesed long biceps tendon in the bicipital groove remained intact. (b) There is a grade 1 retear of the supraspinatus tendon and degenerative changes of the AC joint. (c) There is a grade 1 partial, articular-sided retear of the infraspinatus tendon. (d) The sagittal view confirms the grade 1 supraspinatus retear. (e) The sagittal view shows the grade 1 articular-sided infraspinatus tear has mild interstitial extension. (f) Axial image shows the arthritic AC joint.

The patient has done well postoperatively. At his 3-year-followup, he complained of minimal pain. His constant score was 90, with full, symmetric ranges of motion, but mild weakness in the right supraspinatus muscle. He also showed mild tenderness at the AC joint. CT arthrogram was obtained (Figure 9), which showed re-tear of supraspinatus (without retraction), and articular-sided partial tear of infraspinatus. There is also evidence of AC joint arthritis. Line 4 reflects these changes at the 3-year mark. The patient was very satisfied with the final result and no further intervention was indicated.

**Line 4: RD+3Y AC 0 BT 1 A1 0**

These two examples demonstrate how a small number of lines of 4D code can provide a global view of this patient's history in an efficient and concise manner. There is minimal need to review numerous clinical dictations or operative notes, and communication between surgeons can be simple as well as comprehensive.

## 4. Discussion 

 An exponential increase in the knowledge of pathologic conditions affecting the shoulder has allowed us to define the natural history of various disease processes. Similarly, surgical techniques have evolved to address the uniqueness of each element of the rotator cuff and surrounding structures. The expansion of knowledge and improvement of techniques require a parallel improvement in classification and communication methodology about the shoulder.

Multiple grading and classification systems described in the literature are often complex and cumbersome to use, while only providing a partial view of the shoulder. The 4D code was developed to provide a comprehensive three-dimensional perspective of the shoulder at various time points of treatment. It has distilled the essence of several previously validated classification and grading systems into reproducible alpha-numeric lines of text which is internationally understood. By consolidating previously validated schemes, the basic tenets of an effective classification system—simplicity, accuracy, and applicability—are maintained.

The premise of utilizing such a code to create a three-dimensional image is derived from the same concept of the binary code that computers use to perform their necessary functions. Just as a computer can process a picture from a combination of 0's and 1's, we have established a systematic grouping of letters and numbers to allow the human mind to formulate a complete picture of the shoulder. Additionally, to our knowledge, this is the first description system in the literature that incorporates the dimension of time, which is a progressive and necessary step forward.

Its reproducibility and simplicity permit communication between physicians, therapists, and other members of the care team. Its flexibility and adaptability should have wide appeal. Our experience with the 4D code thus far has shown providers of all levels have been able to easily learn, apply, and clearly communicate with the system. Ultimately, it is a significant step forward in how shoulder pathology is evaluated, documented, and discussed. 

## 5. Conclusion

 The 4D code is developed to provide a concise yet comprehensive view of the shoulder pathoanatomy. It provides a three-dimensional picture and incorporates the element of time. It consolidates previously validated classification and grading systems into lines of alpha-numeric text that is easily conveyed. Its simplicity and completeness aid patient care between members of a care team as well as communication between surgeons internationally. We think the wider adoption of the 4D code can be a large step forward in the documentation of shoulder pathology and the exchange of this information. 

## Figures and Tables

**Table 1:** Subscapularis grading system based on the Lafosse classification [4].

Type	Descriptions
1	Partial tear of superior third
2	Complete tear of superior third
3	Complete tear of superior two-thirds
4	Complete tear, head concentric, fatty infiltration 1–3
5	Complete tear, head eccentric, fatty infiltration > 3

**Table 2:** Samilson classification of glenohumeral arthritis [8].

Stages	Descriptions
1	Osteophyte < 3 mm
2	Osteophyte 3–6 mm
3	Osteophyte > 6 mm

**Table 3:** SLAP lesion as classified by Synder et al. [16] and Maffet et al. [17].

Types	Descriptions
1	Degenerative, frayed superior labrum with intact biceps anchor.
2	Biceps anchor detachment from the superior glenoid tubercle.
3	Bucket-handle tear of the labrum with an intact biceps anchor.
4	Bucket-handle tear of the labrum that extends into the biceps tendon.
5	Anterior-inferior Bankart that extends superiorly to include an unstable biceps anchor
6	Unstable flap tear of the labrum with a detached biceps anchor.
7	Superior labral detachment with extension into the middle glenohumeral ligament.

**Table 4:** Ellman classification for partial thickness rotator cuff tears [4].

Grades	Descriptions
1	Partial tear < 3 mm deep
2	Partial tear 3–6 mm deep; depth not
	exceeding one-half of the tendon
	thickness
3	Partial tear > 6 mm deep

**Table 5:** Fatty infiltration according to Goutallier classification [10].

Stages	Descriptions
0	Normal muscle
1	Fatty streaks
2	Significant fat, but more muscle than fat
3	Equal amount of muscle to fat
4	Fat is greater than muscle

**Table 6:** Summary of the elements of the 4D code with its possible codes and corresponding descriptions.

Components	Classification systems	Variables	Descriptions
Fundamental elements			
Sidedness		R	Right
		L	Left
Time code		D−	Time of presentation
		D	Before surgical repair
		D+	Immediately postop
		D+*n*M	*n *months postop; W for weeks; Y for years
Subscapularis	Lafosse et al. [4]	0, 1–5	(Table 1)
Long head of biceps		BN	Biceps is normal
		BP	Biceps is pathological
		BA	Biceps is absent or tenotomized
		BT	Biceps is tenodesed
Supraspinatus	Patte [7]	0, 1–3	(Figure 4)
Infraspinatus			
Teres minor			
Secondary elements			
AC		AC	AC joint is arthritic
		ACR	s/p AC joint resection
Glenohumeral arthritis	Samilson and Prieto [8]	GH1–3	(Table 2)
Labrum	Synder et al. [16]	SL1–7	SLAP tear (Table 3)
		SLD	Status post SLAP debridement
		SLR	Status post SLAP repair
		L*x*-*y *	Labral detachment from *x* to *y* o'clock
		LD	Status post labral debridement
		LR	Status post labral repair
Tear configuration		U	U-shaped tear
		V	V-shaped tear
		AL	Anterior L-shaped tear with anterior corner detachment
		PL	Posterior L-shaped tear with posterior corner detachement
Partial thickness tear	Ellman [2]	A1–3	Articular-sided tear, (Table 4)
		B1–3	Bursal-sided tear, (Table 4)
		I	Interstitial tear
Calcific tendonitis		C	Presence of calcific tendonitis
Fatty infiltration	Goutallier et al. [10]	G*abcd *	Subscapularis, supraspinatus, infraspinatus, teres minor (Table 5)
Nerve pathology		SSN	Suprascapular nerve injury
		SSNR	Status post SSN release
		AXN	Axillary nerve injury
		LTN	Long thoracic nerve injury
